# Implementation of a Remote Fidelity Oversight Program for a Multi-Site Exercise Intervention

**DOI:** 10.1093/geroni/igab046.1145

**Published:** 2021-12-17

**Authors:** Jennifer Stevens-Lapsley

**Affiliations:** University of Colorado School of Medicine, Aurora, Colorado, United States

## Abstract

The STEP-HI exercise protocol is a supervised, 2 phase, multimodal, high-intensity exercise program that emphasizes resistance training. Exercise sessions are conducted at an exercise facility and occur on two non-consecutive days/week for 6 months. During specified exercises, the exercise interventionist targets the participant’s eight-repetition maximum (8‐RM), defined as the greatest resistance that can be moved 8 times through full range of motion with good form. A rigorous, remote fidelity monitoring program maximizes consistency of the intervention across sites. This fidelity oversight program is a model for future exercise studies because of its unique remote, hierarchical structure. All exercise interventionists are initially certified by written examination and direct observations. Some exercise sessions are also video recorded and reviewed using fidelity checklists. After initial certification, repeated direct observation and video-based verification of fidelity are repeated at prescribed intervals for each interventionist to ensure sustained consistency of implementation across sites.

